# Primary Herpes Simplex Virus Infection Mimicking a Cervical Malignancy in an Immunocompetent Individual

**DOI:** 10.7759/cureus.2753

**Published:** 2018-06-06

**Authors:** Cara Clure, Colleen Rivard

**Affiliations:** 1 Obstetrics, Gynecology and Women's Health, University of Minnesota, Minneapolis, USA; 2 Gynecologic Oncology, University of Minnesota, Minneapolis, USA

**Keywords:** hsv, cervical cancer

## Abstract

Herpes simplex virus (HSV) is a common sexually transmitted infection. Although primary HSV typically presents with ulcerations, atypical presentations are possible. Only one other case report of HSV manifesting as a cervical mass exists. A 35-year-old immunocompetent female with dysuria was found to have a cervical mass concerning for cancer. She had a history of abnormal pap smears and a loop electrosurgical excision procedure* *(LEEP) with poor follow-up. The patient was taken to the operating room for biopsies and staging and was found to have new vulvar ulcers. The biopsies confirmed an HSV infection with cervical, bladder, and vulvar involvement and were negative for cervical neoplasia. This report examines an atypical presentation of a primary HSV infection and reviews the literature regarding the association of HSV with cervical cancer.

## Introduction

Herpes simplex virus (HSV) is one of the most common sexually transmitted infections. Although ulcerated lesions are typically the first sign of primary genital HSV infections, initial presentation is quite variable [1]. We present a case of an atypical HSV presentation in which the patient was noted to have a cervical mass concerning for cervical cancer. 

## Case presentation

A 35-year-old gravida four para four female initially presented to urgent care with dysuria and was prescribed antibiotics for a presumed urinary tract infection. She had no significant medical history and was not immunocompromised in any way. Three days later, she was seen by her primary care provider for worsening dysuria, pelvic pressure, and two days of abnormal vaginal bleeding. Prior to this, she denied any history of abnormal vaginal bleeding and reported normal menses. Due to the grossly abnormal appearance of her cervix, she was referred to gynecology oncology for evaluation. The patient’s medical and surgical history was remarkable for a history of loop electrosurgical excision procedure (LEEP) in her late twenties for cervical intraepithelial neoplasia (CIN) grade 2/3, ESSURE (Bayer, Whippany, New Jersey) placement, and chronic tobacco use. She had an inconsistent follow-up after her LEEP, reporting a normal last pap smear “years prior". She was sexually active with a new male partner for the last four months and denied any history of sexually transmitted infections, including HSV. On examination, normal external genitalia were noted without any lesions or abnormalities. Speculum exam revealed grossly bloody vaginal discharge and an approximately 4-cm firm, circumferential cervical mass with possible left parametrial involvement. 

**Figure 1:**
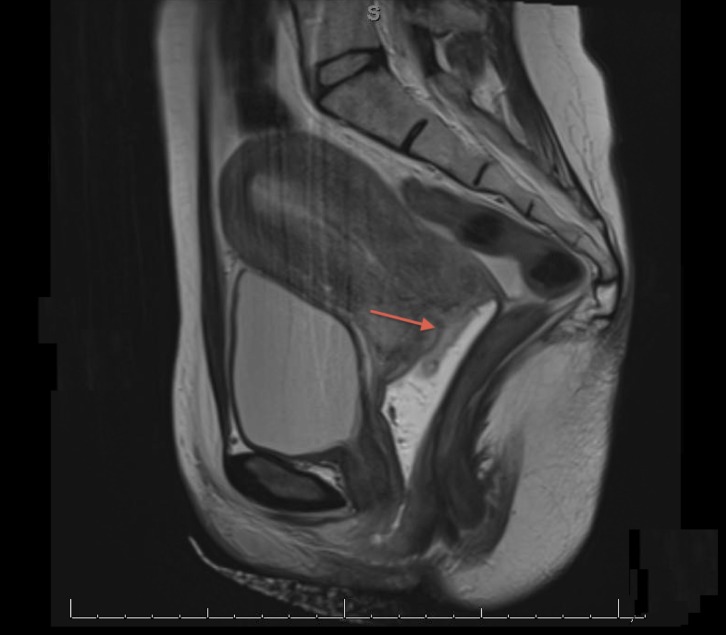
Magnetic resonance image (MRI) of the cervical mass Magnetic resonance imaging, sagittal view. Arrow indicates cervical mass with elevated T2 signal encircling the external cervical os.

An office biopsy of the cervical mass was obtained, and the tissue pathology was found to be negative for malignancy, but remarkable for acute inflammation and cellular changes suggestive of HSV infection. Pap smear results were unremarkable and tested negative for high-risk human papillomavirus (HPV). A magnetic resonance imaging (MRI) scan revealed an elevated T-2 signal concerning for potential neoplasm at the outer surface of the cervix, which encircled the cervical os. Mildly prominent right inguinal lymph nodes were also noted on MRI (Figure 1). Due to the ongoing concern for possible cervical malignancy, the patient was taken to the operating room for an exam under anesthesia, additional cervical biopsies, cervical and vulvar cultures, and cystoscopy with bladder cytology. The exam was remarkable for multiple, new external vulvar lesions concerning for HSV, which were cultured. There was a redemonstration of the 4-cm cervical mass with obliteration of the vaginal fornices and without parametrial involvement. The mass was noted to be friable and ulcerated. Normal bladder mucosa without lesions or invasion and a large amount of bladder debris were seen on cystoscopy. Due to suspected HSV, the patient was started on valacyclovir 1000 mg twice daily for 10 days. All cervical biopsies, including deep biopsies, returned negative for malignancy and showed ulceration and inflammation. Cervical, vulvar, and urine cultures all returned positive for HSV.

The patient was seen approximately two weeks later for follow-up, at which time she reported resolution of her symptoms. Pelvic examination showed no active vulvar lesions and a slightly erythematous cervix with near resolution of the cervical mass and normal vaginal fornices. She was counseled regarding her diagnosis of HSV. Follow-up with a general obstetrician-gynecologist was recommended in one month with a repeat pap smear in two to three years, due to concern for inadequate testing secondary to the presence of the cervical inflammation. It was recommended that she undergo human immunodeficiency virus (HIV) testing; however, the patient declined.

## Discussion

We present the case of a young female who presented with a cervical mass as her initial manifestation of a primary genital HSV infection. Only one other case report of primary HSV presenting as a cervical mass exists. In that case report, by Tomkins et al., the patient, who also was immunocompetent, never developed cutaneous manifestations of HSV [2]. Our patient initially presented without cutaneous lesions; however, she developed cutaneous vulvar ulcers while undergoing further work-up for cervical cancer.    

Although classically primary genital HSV infections present with cutaneous ulcerative lesions, initial presentation is variable. The average incubation period after HSV exposure is four days. Patients can present with systemic symptoms (i.e., fevers, myalgias, malaise, and headache), vulvar pain, dysuria, ulcerations, or tender lymphadenopathy. However, other rarer presentations exist, including acute urinary retention and lumbosacral radiculopathy, the latter of which is primarily seen in immunocompromised patients [1]. The most common cervical abnormalities associated with HSV infections are friability, ulcerative lesions, and cervicitis [3]. Our case report shows that a cervical mass is an additional cervical manifestation of HSV in a presumed immunocompetent individual. Masses have been more commonly described in immunosuppressed patients, specifically in patients with poorly controlled HIV (CD4 counts 326 cells/uL and below) [4].  In this patient population, atypical presentations have been shown to delay proper diagnosis and treatment [5]. 

Despite our patient’s risk factors and presentation, no cervical neoplasia was identified. The possible association of HSV and cervical dysplasia has been hypothesized for decades. While human papillomavirus (HPV) is clearly linked to cervical cancer, the role of HSV is less clear. HSV may facilitate carcinogenesis in HPV-infected cervical cells, but studies continue to produce inconsistent results. A large prospective cohort study of 604 women with cervical cancer and 2,980 matched controls showed no significant association of HSV with cervical cancer (OR 1.1; 95% CI, 0.8-1.5) [6]. A meta-analysis by Cao et al. analyzed 20 longitudinal nested or traditional case-control studies (including the previously described study by Dahlstrom et al.) and found a positive association between HSV-2 infection and the risk of cervical cancer, but no significant association was seen when only the six nested case-control studies were analyzed. Given that the nested study design is more likely to eliminate bias, the study concludes that the evidence is inadequate to support a link between HSV-2 and cervical cancer risk [7].

In summary, a cervical mass mimicking cancer is an atypical presentation of HSV, which can be seen even in immunocompetent patients. It is important to recognize this possible manifestation in order to correctly diagnose and properly treat patients. While our patient did not have cervical neoplasia, the link between HSV and cervical cancer remains controversial, although literature tends to support no association. 

## Conclusions

Although uncommon, primary genital herpes can manifest as a cervical mass, imitating cervical cancer. This is only the second such case of a primary HSV infection presenting as a cervical mass concerning for cervical cancer reported in an immunocompetent individual. This is a rare presentation for primary HSV infection.
